# Improved vector control of *Triatoma infestans* limited by emerging pyrethroid resistance across an urban-to-rural gradient in the Argentine Chaco

**DOI:** 10.1186/s13071-021-04942-9

**Published:** 2021-08-28

**Authors:** María Sol Gaspe, Marta Victoria Cardinal, María del Pilar Fernández, Claudia Viviana Vassena, Pablo Luis Santo-Orihuela, Gustavo Fabián Enriquez, Alejandra Alvedro, Mariano Alberto Laiño, Julieta Nattero, Julián Antonio Alvarado-Otegui, Natalia Paula Macchiaverna, María Carla Cecere, Héctor Freilij, Ricardo Esteban Gürtler

**Affiliations:** 1 Laboratorio de Eco-Epidemiología, Facultad de Ciencias Exactas y Naturales, Universidad de Buenos Aires, Ciudad Universitaria, C1428EHA Buenos Aires, Argentina; 2 Instituto de Ecología, Genética y Evolución de Buenos Aires, Consejo Nacional de Investigaciones Científicas y Técnicas, Ciudad Universitaria, C1428EHA Buenos Aires, Argentina; 3 Washington State University, Paul G. Allen School for Global Animal Health, Allen Center, 1155 College Ave., Pullman, WA 99164 USA; 4 Centro de Investigaciones de Plagas e Insecticidas (CIPEIN, CONICET/UNIDEF/CITEDEF), Juan Bautista La Salle 4397, Villa Martelli, CP 1603 Buenos Aires, Argentina; 5 Universidad Nacional de San Martín, Buenos Aires, Argentina; 6 Cátedra de Química Analítica Instrumental, Facultad de Farmacia y Bioquímica, Universidad de Buenos Aires, Buenos Aires, Argentina; 7 Servicio de Parasitología, Hospital de Niños Ricardo Gutiérrez, Instituto Multidisciplinario de Investigación en Patologías Pediátricas, CONICET-GCBA, Buenos Aires, Argentina

**Keywords:** Gran Chaco, Sustainability, *Triatoma infestans*, Urban, Vector control, Pyrethroid resistance

## Abstract

**Background:**

The sustainable elimination of *Triatoma infestans* in the Gran Chaco region represents an enduring challenge. Following the limited effects of a routine pyrethroid insecticide spraying campaign conducted over 2011–2013 (first period) in Avia Terai, an endemic municipality with approximately 2300 houses, we implemented a rapid-impact intervention package to suppress house infestation across the urban-to-rural gradient over 2015–2019 (second period). Here, we assess their impacts and whether persisting infestations were associated with pyrethroid resistance.

**Methods:**

The 2011–2013 campaign achieved a limited detection and spray coverage across settings (< 68%), more so during the surveillance phase. Following community mobilization and school-based interventions, the 2015–2019 program assessed baseline house infestation using a stratified sampling strategy; sprayed all rural houses with suspension concentrate beta-cypermethrin, and selectively sprayed infested and adjacent houses in urban and peri-urban settings; and monitored house infestation and performed selective treatments over the follow-up.

**Results:**

Over the first period, house infestation returned to pre-intervention levels within 3–4 years. The adjusted relative odds of house infestation between 2011–2013 and 2015–2016 differed very little (adj. OR: 1.17, 95% CI 0.91–1.51). Over the second period, infestation decreased significantly between 0 and 1 year post-spraying (YPS) (adj. OR: 0.36, 95% CI 0.28–0.46), with heterogeneous effects across the gradient. Mean bug abundance also dropped between 0 and 1 YPS and thereafter remained stable in rural and peri-urban areas. Using multiple regression models, house infestation and bug abundance at 1 YPS were 3–4 times higher if the house had been infested before treatment, or was scored as high-risk or non-participating. No low-risk house was ever infested. Persistent foci over two successive surveys increased from 30.0 to 59.3% across the gradient. Infestation was more concentrated in peridomestic rather than domestic habitats. Discriminating-dose bioassays showed incipient or moderate pyrethroid resistance in 7% of 28 triatomine populations collected over 2015–2016 and in 83% of 52 post-spraying populations.

**Conclusions:**

The intervention package was substantially more effective than the routine insecticide spraying campaign, though the effects were lower than predicted due to unexpected incipient or moderate pyrethroid resistance. Increased awareness and diagnosis of vector control failures in the Gran Chaco, including appropriate remedial actions, are greatly needed.

**Graphical abstract:**

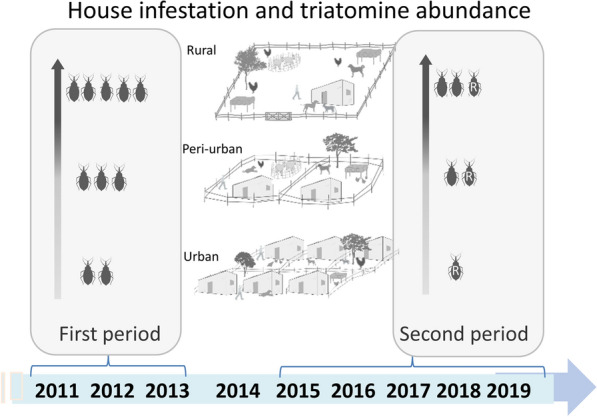

**Supplementary Information:**

The online version contains supplementary material available at 10.1186/s13071-021-04942-9.

## Background

More than a century after its discovery, Chagas disease still is one of the most important neglected tropical diseases (NTD) in Latin America, where it affects 6–7 million people [1]. The transmission of *Trypanosoma cruzi*, the causative agent of Chagas disease, is mainly mediated by triatomine bugs and vertically from mother to newborn, but also can occur through blood transfusion or organ transplant, and by ingestion of contaminated foodstuff. The epidemiological features of Chagas disease gradually changed from being almost exclusively associated with poor rural settings to causing worldwide health concerns [2, 3].

Several regional initiatives to curb Chagas disease transmission were launched in the 1990s, all of which included a strong triatomine control component [4]. In the Southern Cone countries, the Gran Chaco ecoregion cuts across sections of Argentina, Bolivia and Paraguay, and is a well-known hotspot of chronic poverty and several NTDs including Chagas disease [5]. There, the prevalence of house infestation with *Triatoma infestans* has historically been high [6–8] and remained so in some (e.g., [9, 10]) but not across all rural areas (e.g., [11]). Ecological, socio-demographic, political and economic constraints hinder vector control success in the Gran Chaco, including the lower effectiveness of pyrethroid insecticides in peridomestic structures, limited coverage of control actions, disperse rural settlements with limited access, operational and resource limitations, occurrence of sylvatic foci, among others [12, 13]. This epidemiological scenario gained more complexity over the 1990s with the emergence of pyrethroid resistance in *T. infestans* and associated control failures across northern Argentina and Bolivia [14–18]. The issue of rapid recovery of triatomine populations following insecticide spraying campaigns cuts across the major triatomine vector species, such as *Triatoma dimidiata*, *Triatoma brasiliensis*, *Rhodnius ecuadoriensis* and *Rhodnius prolixus* (e.g., [19–23]).

Vector-borne transmission of *T. cruzi* mediated by several triatomine species also occurs in urban and peri-urban environments (e.g., [24–27]). Key obstacles to triatomine control success in large urban and peri-urban areas include poor levels of household participation combined with limited access to premises [28, 29], large number of houses albeit in close proximity, and eventual violence and security issues. Chagas disease is no longer a rural health issue, and its current scope poses new challenges to vector control efforts.

Insecticide-based control programs designed to suppress domestic infestations with *T. infestans* in rural villages located in the dry (Amamá, Santiago el Estero) and humid Argentine Chaco (Pampa del Indio, Chaco) were successful only if sustained consistently over extended periods (e.g., [9, 11, 30–32]). In Avia Terai municipality (Chaco), Chagas disease vector control teams conducted a district-wide, routine insecticide spraying campaign to suppress triatomine infestations over 2011–2013. In spite of sizable control efforts, house infestations persisted for undefined reasons, and local authorities requested additional interventions. Drawing from our previous experience, we designed and implemented a rapid-impact intervention package over 2015–2019 with the goals of suppressing house infestation with *T. infestans* across the municipality and reducing disease burden in a sustainable manner. The program included community mobilization and school-based interventions at the outset, and was framed on ecohealth principles [33]. Baseline house infestation in 2015–2016 increased across the urban-to-rural gradient from 11.3 to 42.4%, respectively [34]. A simple index of house infestation risk (based on householders’ reports of infestation and availability of peridomestic structures) had maximum sensitivity and negative predictive value but low specificity. Triatomine infection with *T. cruzi* also increased across the gradient from nil to 2.2%, whereas the intensity of domestic vector–human contact was uniformly high [35].

Here, we assess the impacts of the interventions implemented over the first (2011–2013) and second periods (2015–2019) on house infestation across rural, peri-urban and urban settings of Avia Terai. We sought to identify the reasons for lower-than-expected effects on infestation over the first period; whether the improved coverage of interventions (triatomine detection and supervised insecticide spraying) combined with community mobilization exerted greater impacts than the preceding effort; and assess the post-intervention performance of the index of house infestation risk. A key question was whether the persisting levels of infestation were associated with pyrethroid resistance, although there were little grounds for any expectation given the sparse history of local pyrethroid applications to suppress triatomines. Based on the background evidence reviewed above, we hypothesized that (i) the limited effects on house infestation over 2011–2013 were related to limited insecticide spray coverage and non-systematic vector surveillance; (ii) peridomestic infestations would prevail over domestic foci across the gradient due to the poorer performance of pyrethroids outdoors; and (iii) house infestation prevalence would increase across the urban-to-rural gradient (likewise before interventions) because urban areas had fewer peridomestic structures and lower domestic animal availability. This study may be the first to provide a detailed assessment of the relative effectiveness of insecticide spraying on house infestation across urban-to-rural settings of a traditionally endemic district.

## Methods

### Study area

The intervention program was implemented in Avia Terai municipality (26° 42′ S 60° 44′ W), Chaco province, north-eastern Argentina. The main features and map of the study area were reported elsewhere [34]. The urban setting had 1409 inhabited dwellings arranged in a 10-by-10 block matrix as of 2016. Peri-urban settings included 575 inhabited dwellings distributed in four recent peri-urbanization areas and four established neighbourhoods. The rural setting comprised 308 inhabited houses as of 2015 (including 34 borderline houses from neighbouring districts).

### Study design

Over the first period of interventions (2011–2013), vector control personnel from various jurisdictions assessed house infestation by means of timed-manual searches with a dislodging aerosol (0.2% tetramethrin; Espacial, Argentina) (hereafter denoted as timed-manual searches), and then performed house spraying with suspension concentrate pyrethroid insecticides (beta-cypermethrin: Sipertrin, Chemotecnica, Argentina; deltamethrin: K-Othrine, Bayer, Argentina, or alpha-cypermethrin, BASF, Argentina) using standard doses. This large endeavour also included communicational aspects [36, 37] and diagnosis and etiological treatment of *T. cruzi*-seropositive residents of Avia Terai municipality. House inspections for triatomines mainly targeted neighbourhoods or settings whose dwellers notified triatomine presence or requested control actions, especially in urban and peri-urban settings, according to local vector control personnel. Searches for triatomines and insecticide spraying were implemented in rural areas in 2011, in peri-urban areas in 2012, and in urban areas both in 2012 and 2013. Rural and peri-urban insecticide treatments included almost every inspected house, whereas only half of the inspected urban dwellings were sprayed.

Some partial interventions performed after the initial treatment were not included in estimates of house infestation prevalence or treatment coverage to avoid counting them twice, as follows. In 2012, houses from a previously infested rural community were re-inspected and re-sprayed with pyrethroids (*n* = 122); in 2013 and early 2015, two and four rural infested houses were inspected and sprayed, respectively. In late 2013, 2014 and early 2015, 96 urban and 7 peri-urban houses were selectively sprayed with pyrethroid insecticide in response to householders’ requests, including five urban houses positive for *T. infestans*. Some houses inspected for triatomines in peri-urban (*n* = 34) and urban areas (*n* = 125) in 2011 that were not subsequently sprayed with insecticide were considered untreated.

We computed house inspection coverage, house infestation prevalence, and coverage of insecticide spraying over 2011–2013 from the records of vector control interventions kept by the Chagas disease control program of Chaco Province. Inspection and spray coverage were calculated relative to the number of inhabited houses we registered in 2015–2016.

The intervention program launched in 2015 included an initial phase of community mobilization and school-based interventions to engage householders and promote community-based vector surveillance at the outset (to be reported separately); a baseline assessment of house infestation with triatomines, and a new house spraying with pyrethroid insecticides in urban, peri-urban and rural settings of Avia Terai following established practices. Because no specific guidelines for triatomine control actions were in place for peri-urban or urban settings and the scale of operations exceeded the available resources, we initially used a selective spray protocol and then adapted actions according to the recorded response (see below). Operations were coordinated with local municipal, education and hospital officials, and gradually scaled up district-wide starting in rural areas up to covering peri-urban neighbourhoods and urban blocks over a 6-month period (Table 1).

**Table 1:** Type of triatomine survey and house spraying by type of environment and date over the second period, Avia Terai 2015–2019

Years post-spraying (date)	Environment	Type of vector survey	Type of spraying	Type of searches between surveys
0 (Feb 2016)	Urban	Stratified sample^a^	Infested + adjacent	–
0 (Dec 2015–Mar 2016)	Peri-urban	Stratified sample^a^	Infested + adjacent	–
0 (Oct 2015)	Rural	Complete	Complete	–
1 (Mar–May 2017)	Urban	Stratified sample^b^	Infested blocks	HN
1 (Mar–May 2017)	Peri-urban	Complete	Infested blocks	HN
1 (Nov 2016)	Rural	Complete	Focal	HN
2 (May 2018)	Peri-urban	Complete	Infested blocks	HN
2 (Dec 2017)	Rural	Selective^c^	Focal	Selective^c^, HN
3 (Dec 2018)	Rural	Incomplete	Incomplete	HN
4 (Nov 2019)	Rural	Complete	No	HN

*HN* householder notification of the presence of triatomines in the dwellings

^a^All high-risk houses according to the risk stratification index, and 30% of low-risk houses

^b^All high-risk houses according to the risk stratification index, 30% of low-risk houses, and all houses within an infested block

^c^Including inhabited houses infested with *T. infestans* in the preceding survey

Householders were explained the aims of the project at the outset (2015–2016), and invited to participate and provide oral consent. All buildings were identified with a unique code. Rural houses were georeferenced using a GPS (Garmin Legend) and identified with a numbered aluminium plate located near the front door. Peri-urban houses not arranged in blocks, and urban and peri-urban blocks were also georeferenced. The baseline survey of house infestation with triatomines, conducted across the gradient between October 2015 and March 2016, used a stratified sampling strategy [34]. A rapid risk assessment (based on householders’ reports of triatomine presence after showing them dry specimens of *T. infestans*, *Triatoma sordida* and other Reduviidae, and/or the existence of peridomestic structures housing domestic animals) allowed us to score all inhabited houses according to their putative chances of being infested at that time regardless of previous status. All high-risk houses were inspected for triatomines using timed-manual searches, whereas a systematic sample (30%) of low-risk houses was surveyed using the same procedures [34].

Skilled vector control personnel from Chagas disease control programs accompanied by at least one member of the research team conducted vector searches and insecticide applications. Searches were performed in each domicile and peridomestic structure by one person for 15 min in each site. Closed houses were re-visited at least once or twice in order to increase coverage. Uninhabited houses and public buildings were inspected for triatomines only if they were occasionally occupied or if domestic animals rested inside. Triatomines collected by householders and during insecticide spraying were used to assess house infestation status by any method.

Immediately after triatomine surveys, vector control personnel sprayed houses with suspension concentrate beta-cypermethrin (Sipertrin, Chemotecnica, Argentina) using a simple dose (50 mg/m^2^) in domiciles and a double dose in peridomiciles (100 mg/m^2^) to ensure longer-lasting effects [38]. Insecticide, spray gear and dislodging aerosol were provided by the Chagas disease control program. Rural houses received a full-coverage, community-wide treatment. In urban and peri-urban settings, only infested houses and adjacent units were sprayed with insecticide considering their low infestation levels, large frequency of house units, time and budget constraints, and insecticide availability.

Periodic searches were performed after control interventions to assess house infestation status and to identify putative reinfestation foci or vector control failures at 1 year post-spraying (1 YPS, November 2016–May 2017), 2 YPS (December 2017–May 2018) and 4 YPS (November 2019) (Table 1). Post-spraying surveys aimed to achieve full coverage in established peri-urban neighbourhoods and rural settings, both of which had displayed high house infestation prevalence. All inhabited urban houses were scored for the post-intervention risk index as in the baseline survey, and triatomine searches were performed in all high-risk houses and in a systematic sample (30%) of low-risk houses. In addition, all houses located in blocks harbouring at least an infested house were additionally inspected for triatomines. All infested houses were sprayed with insecticide after each vector survey using the same procedures described above. In view of the occurrence of persistent block-level infestations at 1 YPS, every house located in an infested block was sprayed with insecticides in subsequent urban and peri-urban surveys (Table 1). In rural areas and peri-urban settings not arranged in blocks, only the infested houses were selectively sprayed during the surveillance phase (i.e., focal spraying). The follow-up of infested rural houses at 2 YPS was limited to infested houses during the preceding survey and remained incomplete due to persistent rainy weather. Similarly, shortage in vector control personnel (at 3 YPS) and insecticide (at 4 YPS) determined that monitoring of house infestation and treatment in rural areas remained incomplete or nil, respectively.

For community-based vector surveillance, householders were encouraged to collect any triatomine sighted using safe procedures; keep them in a plastic bag we provided them, and deliver it to the nearest school, healthcare post, the local hospital, or local vector control referent. Local personnel inspected houses whose dwellers collected any triatomine or notified its occurrence (with no triatomine handed in) over the period elapsed between successive surveys. When house infestation with *T. infestans* was confirmed, all structures pertaining to an infested house were sprayed with insecticides as described above.

Triatomines were kept in labelled plastic bags identified by collection site, date and sampling method, and transported to the field laboratory where they were identified taxonomically and counted according to species, stage, or sex as described elsewhere [39]. A sample of adult triatomines collected in each survey was selected for screening of pyrethroid resistance status (see below); the remaining insects were kept at −20 °C.

### Pyrethroid resistance

A sample of rural and peri-urban populations of *T. infestans* collected over 2015–2016 (baseline) was reared separately by collection site to assess their mortality levels in pyrethroid resistance bioassays conducted at Centro de Referencia de Vectores (Punilla, Córdoba, Argentina). Insects were blood-fed through the open extreme of insect rearing jars covered with voile applied onto the pigeon’s skin. This proceeding was carried out weekly according to the protocol approved by the Institutional Animal Care and Use Committee of CIPEIN (National System of Bioterium Registry Number: 1572/155).

Local triatomine populations were grouped by spatial proximity for a rapid screening, including 31 (26%) rural houses and nine (15%) peri-urban houses grouped in six and two pools, respectively, each of which had 3–10 houses. One rural and two peri-urban populations collected at baseline were tested individually. Bug collection sites from which we recovered less than 10 eggs were excluded from the analyses.

A sample of *T. infestans* populations collected over 2016–2019 across environments were individually tested for deltamethrin resistance status at CIPEIN. The tested samples represented 13–38% of infested houses as determined by any method.

Screening tests at both laboratories followed the same procedures [40, 41] with additional details provided elsewhere [18]. The dorsal abdomens of individual first-instar nymphs of *T. infestans* were treated topically with a 0.2 μl acetone solution of technical-grade deltamethrin (99.0%, Ehrestorfer, Augsburg, Germany) containing a discriminating dose (0.01 mg/ml, equivalent to 0.2 ng/insect). This assay determined whether the bug population was resistant or susceptible to deltamethrin. Laboratory-reared colonies of *T. infestans* were used as a negative control (pyrethroid-susceptible) and positive control (a pyrethroid-resistant strain from Salta, Argentina which carries the *kdr* gene); both control tests invariably showed 100% and 0% mortality, respectively. Mortality was evaluated 24 h after deltamethrin application. We aimed to achieve three replicates of at least 10 insects each for each study bug population. The pyrethroid-resistance status of each bug population was scored according to bioassay mortality: susceptible (bug mortality greater than 90%), incipient resistance (76–90%), moderate resistance (45–75%) and high resistance (less than 45%). If a pool resulted susceptible, then each house included was considered susceptible. When the pool was scored as resistant, only the house with the highest number of eggs included in the sample was considered pyrethroid-resistant on conservative grounds. For triatomine populations showing low mortality in discriminating-dose bioassays, we tested a 5× greater concentration of deltamethrin depending on the availability of first-instar nymphs.

### Data analysis

Public buildings, abandoned houses and recent peri-urbanization areas were excluded from infestation analyses because none of them were found infested with triatomines at baseline [34] and thereafter. Only established peri-urban neighbourhoods were considered for peri-urban infestation analyses. A house was considered infested if any live *T. infestans* was found by timed-manual searches, unless explicitly noted. Colonization was defined as the presence of at least one live nymph of *T. infestans*. A house or block was considered persistently infested if it was found infested in two successive surveys despite any insecticide treatment in the intervening period. Houses found infested between surveys conducted at *t* − 1 and *t* (and sprayed with insecticide) were included as infested at time *t* to compute overall infestation prevalence, assuming that in the absence of treatment, they would have remained infested until *t*.

We computed a house-level average of bug abundance as the total number of live triatomines collected by timed-manual searches (using one person-hour) at a house divided by the number of suitable sites inspected for triatomines within that house. These data were only available for 2015–2019. Suitable sites included frequently infested ecotopes, such as domiciles and peridomestic habitats (kitchens/storerooms/granaries, chicken coops/nests, corrals); dog resting sites and piled materials were also included due to their relevance in the urban area. Sites from other rarely infested ecotopes (bathrooms, trees with chickens, ovens, among others) were only considered when they contained any potential host or any triatomine was caught. The computation of averaged bug abundance is more representative of bug numbers across all inspected sites with the goal of estimating intervention effects; it differs from our previous estimates of pre- or post-intervention median bug abundance, which were restricted to infested sites only [34]. Relative bug abundance at each house was transformed to log_10_ (bug abundance + 1) for comparisons between environments and surveys. The number of inhabited urban houses at baseline slightly differ (*n* = 4) from our previous results [34] as a minor duplication was found in the database.

We were able to re-identify 99 rural houses (first inspected for triatomines and sprayed in 2011 and subsequently re-inspected in 2015) by means of head of households’ names for a detailed assessment of insecticide spraying effects.

Cochran–Mantel–Haenszel (CMH) *χ*^2^ tests and summary OR were used to examine the association between pre- and post-intervention house infestation stratified for type of environment for both study periods; homogeneity *χ*^2^ tests examined whether the effects differed significantly across environments. Similar analyses were made separately for domestic and peridomestic habitats. Differences in log-transformed bug abundance between surveys were assessed through non-parametric Mann–Whitney tests. Agresti–Coull 95% confidence intervals for proportions were calculated for infestation prevalence. Statistical analyses were performed in Stata 14.2 [42].

The associations between house infestation or relative bug abundance at 1 YPS (response variables) and selected explanatory variables (baseline house infestation with *T. infestans*, insecticide spraying, baseline house risk index and type of environment) were tested through logistic and negative binomial regression analysis, respectively. No multicollinearity among explanatory variables was detected (variance inflation factors < 1.8 for every variable). The interactions between type of environment and each explanatory variable were assessed, and those with significant effects at the 5% level were retained in the model. The overall fit of the logistic model was assessed by the Hosmer–Lemeshow test (pooling the data in 10 groups) and the area under the receiver operating curve (ROC).

Global point pattern analyses of house infestation at 1 YPS were performed at house level (in the rural area), at block level (in the urban area), and blocks or houses (in the peri-urban neighbourhoods) according to the spatial arrangement of houses. Global spatial analyses of house/block infestation were performed using the K-function implemented in Programita [43] using random labelling to test the null hypothesis of random events among the fixed spatial distribution of all houses (or blocks). The selected cell size was 300 m for the rural environment and 50 m for urban or peri-urban analyses; the maximum distance was set at 15 and 0.6 km, respectively (i.e., half of the dimension of the area). Monte Carlo simulations (*n* = 999) were performed, and the 95% confidence envelope was calculated with the 2.5% upper and lower simulations.

## Results

### Intervention effects over the first period (2011–2013)

**Fig. 1:**
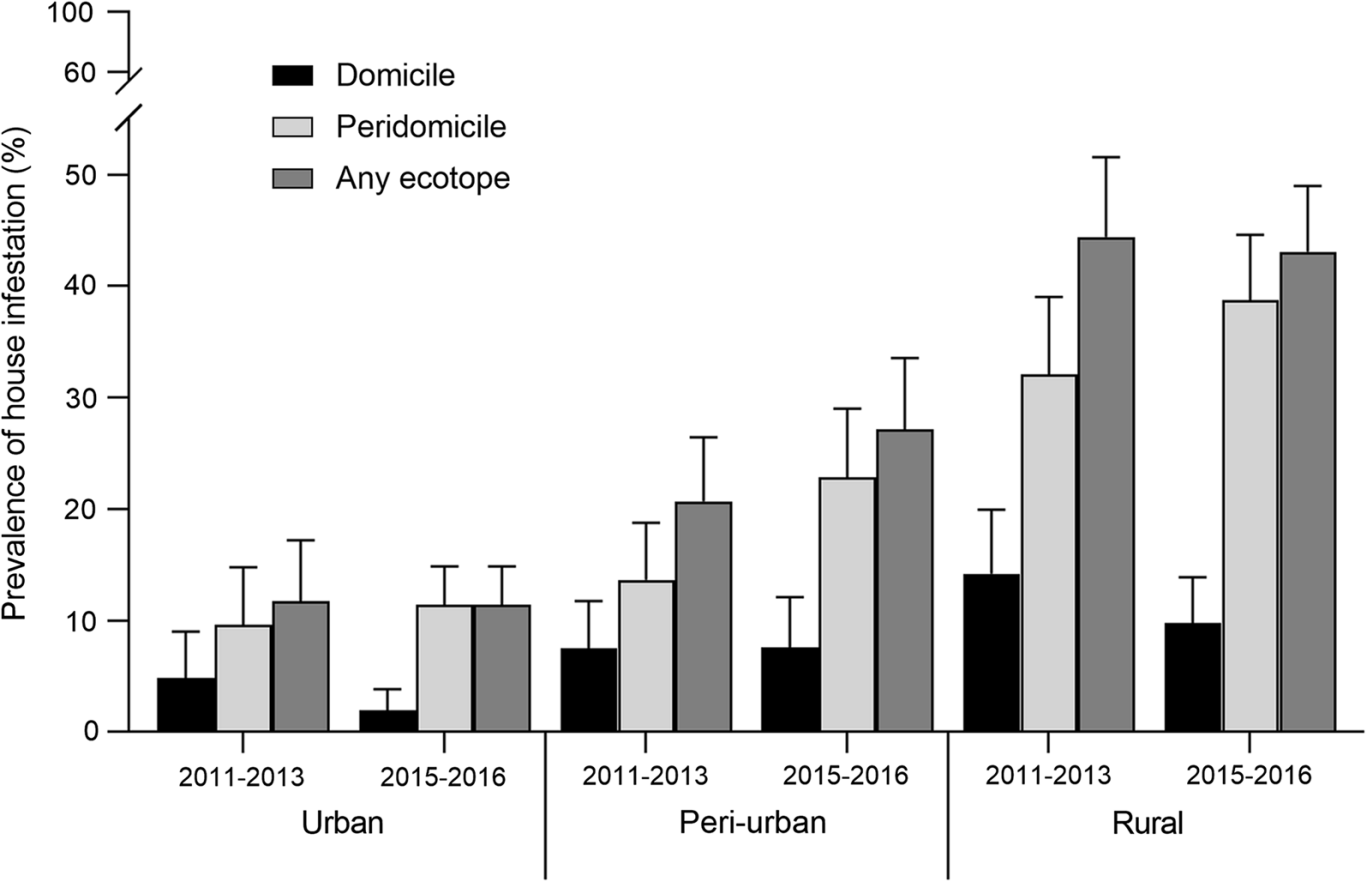
House infestation with *T. infestans* over 2011–2013 and 2015–2016 (as determined by timed-manual searches) according to habitat (domestic, peridomestic) across the urban-to-rural gradient, Avia Terai, 2011–2016. Whiskers show Agresti–Coull 95% confidence intervals

House infestation with *T. infestans* over 2011–2013 increased steadily and significantly from 11.8 to 40.0% across the urban-to-rural gradient, respectively (*χ*^2^ = 43.4, *df* = 2, *P* < 0.001) (Fig. 1). House inspection and treatment coverage were more limited in urban (13.3% and 6.7%, respectively, *n* = 1409) than in rural (61.7 and 61.4%, *n* = 308) or peri-urban (73.9% and 67.8%, *n* = 307) environments (Table 2). Insecticide spray coverage reached 491 houses during the attack phase and 231 during the surveillance phase (total, 722 houses). Infestation was more frequent in peridomestic rather than domestic ecotopes in rural (32.1% versus 14.2%), peri-urban (13.7% versus 7.5%) and urban areas (9.6% versus 4.8%).

**Table 2:** Distribution of house infestation with *T. infestans* by timed-manual searches according to type of environment and survey, Avia Terai, Chaco, 2011–2019

Years post-spraying (Date)	Environment	% of inspected houses (no. registered)	% of infested houses (no. inspected)^c^	% of sprayed houses (no. sprayed)^d^
(2011–2013)^b^	Urban	13.3 (1409)^a^	11.8 (187)	6.7 (94)^a^
	Peri-urban	73.9 (307)^a^	20.7 (227)	67.8 (208)^a^
	Rural	61.7 (308)^a^	40.0 (190)	61.4 (189)^a^
0 (2015–2016)	Urban	29.2 (1409)	11.4 (412)^e^	7.4 (104)
	Peri-urban	68.4 (307)	27.1 (210)^e^	34.9 (107)
	Rural	89.6 (308)	42.4 (276)^e^	87.3 (269)
1 (2016–2017)	Urban	63.9 (1455)	2.2 (930)	21.4 (311)
	Peri-urban	85.8 (323)	17.0 (277)	42.4 (137)
	Rural	87.7 (310)	21.7 (272)	20.3 (63)
2 (2017–2018)	Peri-urban	86.7 (338)	6.1 (293)	16.3 (55)
	Rural	17.0 (305)	26.9 (52)	4.9 (15)
4 (2018–2019)	Rural	86.9 (283)	19.5 (246)	2.1 (6)

Baseline: October 2015–March 2016; 2011–2013 (first period)

^a^Computed relative to the number of inhabited houses registered in 2015–2016

^b^Additional insecticide treatments during the surveillance phase included 122 rural houses sprayed in 2012–2013, 6 peri-urban houses in 2014–2015, and 87 urban houses in 2014–2015 (total, 215 houses)

^c^House infestation prevalence was calculated as the sum of infested houses at survey *t* and those found infested between *t* − 1 and *t* relative to the number of houses inspected for triatomines

^d^The percentage of sprayed houses was calculated relative to the number of inhabited houses registered in each survey

^e^Observed infestation prevalence

The prevalence of house infestation stratified for type of environment did not differ significantly between 2011–2013 and 2015–2016, the latter taken as an outcome measure of prior interventions (CMH *χ*^2^ = 1.42, *df* = 1, *P* = 0.23; adj. OR: 1.17, 95% CI 0.91–1.51). Intervention effects were homogeneous across strata (homogeneity *χ*^2^ = 1.4, *df* = 2, *P* = 0.5) including urban (11.8% versus 11.4%), peri-urban (20.7% versus 27.1%) and rural areas (40.2% versus 42.4%) (Fig. 1, Table 2). The relative odds of being infested as of 2015 was three times higher if the house had been infested in 2011 among identified rural houses inspected both in 2011 and 2015 (OR: 3.14, 95% CI 1.30–7.57).

When house infestation was restricted to domestic habitats, differences between periods were statistically significant (CMH *χ*^2^ = 7.5, *df* = 1, *P* = 0.006; OR: 0.56, 95% CI 0.37–0.85) across environments (homogeneity *χ*^2^ = 5.6, *df* = 2, *P* = 0.06). Domestic infestation declined from 4.8 to 0.7% in urban areas and from 14.2 to 9.1% in rural areas, and remained at similar levels (7.5% vs. 6.2%) in peri-urban neighbourhoods (Fig. 1). By contrast, peridomestic infestation increased significantly over time (CMH *χ*^2^ = 6.8, *df* = 1, *P* = 0.009; OR: 1.43, 95% CI 1.09–1.87) across strata (homogeneity *χ*^2^ = 1.78, *df* = 2, *P* = 0.4): in peri-urban areas from 13.7 to 22.9%, in rural areas from 32.1 to 38.8%, and in the urban environment from 9.6 to 10.9%.

**Fig. 2:**
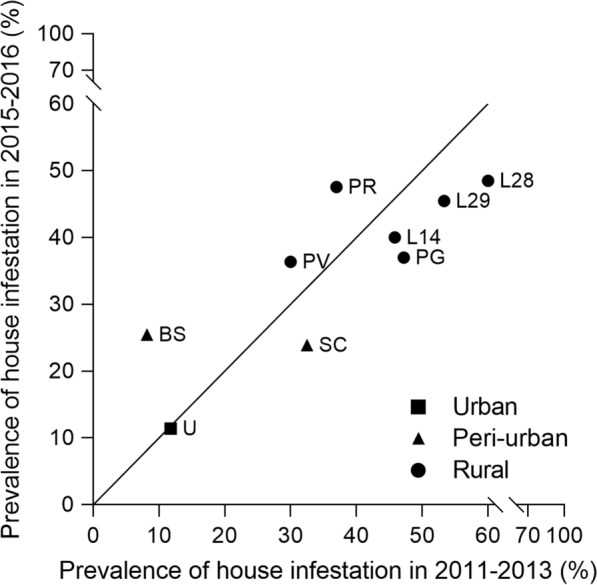
Community-level house infestation with *T. infestans* over 2011–2013 and 2015–2016 in Avia Terai, Chaco. U: urban; SC, BS: peri-urban neighbourhoods; PV, PR, PG, L14, L28, L29: rural communities

The community-level prevalence of house infestation over 2011–2013 correlated positively and significantly with the outcome of 2015–2016 vector surveys (*r* = 0.8, *P* = 0.04, Fig. 2). House infestation levels recovered after 3–4 years in almost every community. One of the most heavily infested rural communities (Pampa Grande, PG) in 2011 (40.4%) displayed high infestation both in 2012 (20.5%), before being re-sprayed with pyrethroid insecticide, and 2015 (37.0%).

### Intervention effects over the second period (2015–2019)

The number of occupied houses slightly decreased from 308 to 283 in rural areas over the 4-year period (2015–2019), and increased from 307 to 338 houses in peri-urban neighbourhoods (2016–2018) and from 1409 to 1455 houses in urban settings (2016–2017) (Table 2). Vacant houses increased in rural areas and remained nearly stable in urban and peri-urban areas over the follow-up. In total, 1479 inhabited houses were inspected for triatomines at 1 YPS (Table 2). Peri-urban and rural houses that failed to be inspected were mainly closed (10.2–10.3%), and a few households refused access to the premises (1.9–4.0%). In the urban area, 25.4% of houses were closed, 1.3% refused to participate, and 9.4% were low-risk houses (a priori excluded from further inspection based on the risk-stratified sampling design). Overall insecticide spray coverage reached 1067 houses (Table 2). In total, 480 inhabited houses were sprayed with insecticide at baseline (2015–2016), reaching a coverage of 7.4%, 34.9% and 87.3% across the urban-to-rural gradient, respectively, and 587 were sprayed over the surveillance phase.

**Fig. 3:**
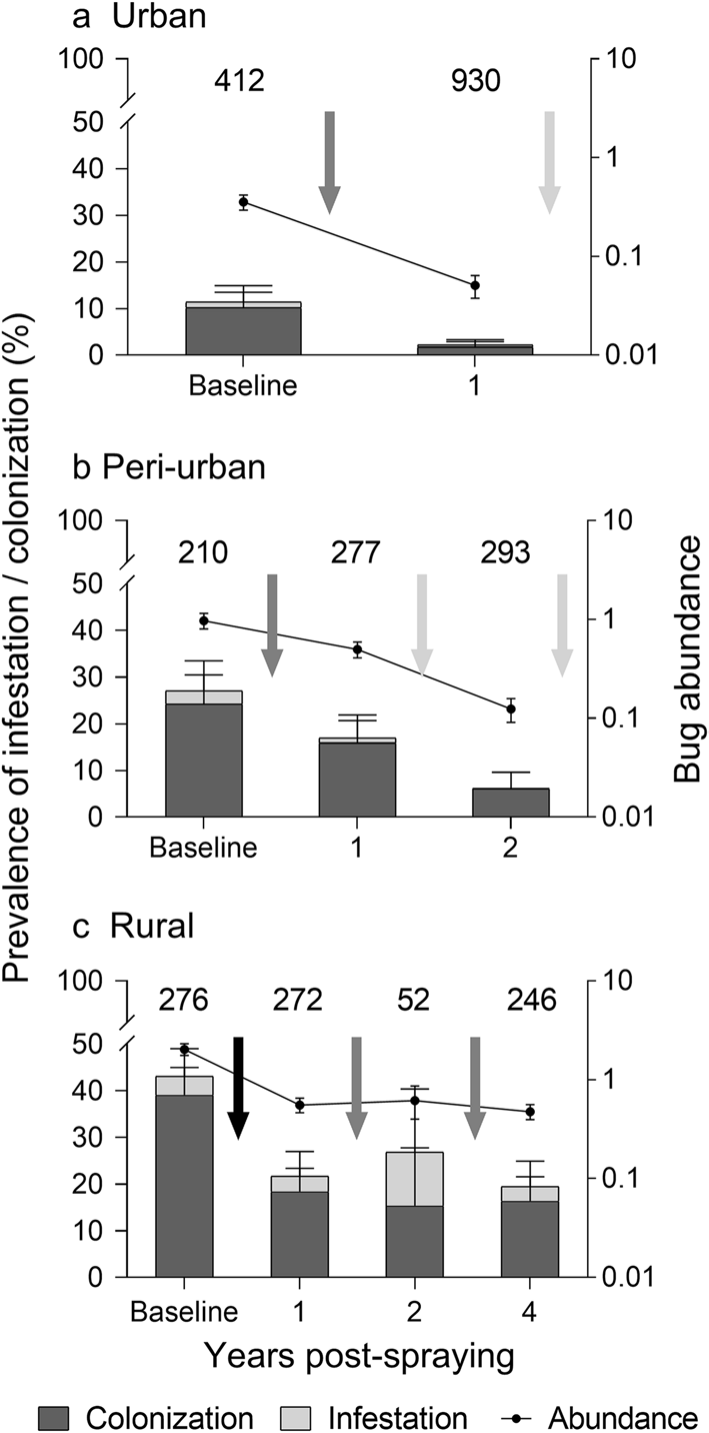
House infestation, colonization and relative bug abundance of *T. infestans* as determined by timed-manual searches in each environment, Avia Terai, Chaco, 2015–2019. The baseline survey was conducted in 2015–2016. Black whiskers show Agresti–Coull 95% confidence intervals for prevalence; grey whiskers show ± SE of mean bug abundance. Numbers above bars represent the number of inhabited houses inspected by timed-manual searches. The arrows represent house spraying with insecticides: black arrow, community-wide spraying; grey arrow, selective spraying of infested houses (including adjacent houses in urban and peri-urban settings); white arrow, selective spraying of every house located in infested blocks

A highly significant decrease in overall house infestation with *T. infestans* as determined by timed-manual searches was observed between 0 and 1 YPS (CMH *χ*^2^ = 70.5, *df* = 1, *P* < 0.001, OR: 0.36, 95% CI 0.28–0.46), with heterogeneous effects across strata (*χ*^2^ = 11.7, *df* = 2, *P* = 0.004) (Fig. 3, Table 2). The relative reduction in urban areas (OR: 0.17, 95% CI 0.10–0.30) was greater than in rural (OR: 0.38, 95% CI 0.26–0.55) or peri-urban areas (OR: 0.55, 95% CI 0.35–0.85). Colonization levels were high across the urban-to-rural gradient (85.0%, 93.6% and 84.7%, respectively) at 1 YPS (Fig. 3). Of 21 infested rural houses treated with insecticides in December 2015 and re-inspected by timed-manual searches 5 months later (in May 2016), 48% were again infested; infestation reached 71% if householder collections were considered.

When considering any triatomine collection method, overall house infestation at 1 YPS increased to 3.9%, 20.2% and 23.9% across the gradient, respectively. Of the 157 infested houses detected by any collection method at 1 YPS, 25 were revealed by householder collections only; most of them occurred in urban areas (56%). Thirty infested houses (63% of them in rural areas) were detected between 0 and 1 YPS surveys based on householder notifications with (*n* = 15) or without (*n* = 15) handing in triatomines; all of the latter were confirmed by timed-manual searches.

When considering infestation prevalence in domiciles versus peridomestic sites, we found that infestation in both habitats decreased at 1 YPS, similarly to the overall trend at house level. However, while this decrease in domiciliary infestation (CMH *χ*^2^ = 28.1, *df* = 1, *P* < 0.001; OR: 0.17, 95% CI 0.08–0.35) was similar across strata (homogeneity *χ*^2^ = 1.9, *df* = 2, *P* = 0.40), the relative reduction in peridomestic infestation (CMH *χ*^2^ = 55.6, *df* = 1, *P* < 0.001; OR: 0.39, 95% CI 0.31–0.51) differed across the gradient (homogeneity *χ*^2^ = 13.7, *df* = 2, *P* < 0.001), with greater reductions in urban areas (OR: 0.18, 95% CI 0.10–0.31) compared to rural (OR: 0.40, 95% CI 0.27–0.59) or peri-urban settings (OR: 0.67, 95% CI 0.43–1.06), similarly to the trend observed at house level.

No low-risk house in urban or peri-urban areas was infested by timed-manual searches or by any other collection method during the 2016–2018 follow-up in which nearly 440 low-risk houses were inspected for triatomines (Table 3). No global spatial aggregation of house- or block-level infestation was detected in urban, peri-urban or rural settings at 1 YPS (Additional file 2: Fig. S1).

**Table 3:** Distribution of house infestation with *T. infestans* by timed-manual searches according to the house risk index^a^, type of environment and survey, Avia Terai, Chaco, 2015–2019

Years post-spraying	Environment	% of infested dwellings (no. inspected)			
		Low-risk	High-risk	No risk data	Total
0	Urban	0.0 (142)	17.4 (270)	– (0)	11.4 (412)
	Peri-urban	0.0 (61)	38.3 (149)	– (0)	27.1 (210)
	Rural	0.0 (23)	46.2 (253)	– (0)	42.4 (276)
1	Urban	0.0 (314)	4.0 (498)	0.0 (118)	2.2 (930)
	Peri-urban	0.0 (66)	22.5 (209)	0.0 (2)	17.0 (277)
	Rural	– (0)	21.7 (272)	– (0)	21.7 (272)
2	Peri-urban	0.0 (59)	9.5 (190)	0.0 (44)	6.1 (293)
	Rural	– (0)	12.8 (47)	– (0)	23.5 (51)
4	Rural	– (0)	15.2 (244)	– (0)	18.4 (245)

Baseline: October 2015–March 2016

^a^House risk index: based on householders’ reports of triatomine presence or the existence of peridomestic structures housing domestic animals

Figure 3 shows that the log-transformed mean bug abundance decreased significantly between 0 and 1 YPS in each environment (Mann–Whitney test, rural: *z* = 5.6, *P* < 0.001; peri-urban: *z* = 2.7, *P* = 0.007; urban: *z* = 7.3, *P* < 0.001). Similarly, log-mean bug abundance restricted to infested houses at baseline decreased significantly at 1 YPS in each environment (Mann–Whitney test, rural: *z* = 10.1, *P* < 0.001; peri-urban: *z* = 5.2, *P* < 0.001; urban: *z* = 7.6, *P* < 0.001).

Peri-urban house infestation at 2 YPS further declined to 6.1% of inspected houses, i.e., a 64.1% relative reduction after insecticide spraying of all houses in a block that had had at least an infested house at 1 YPS (*χ*^2^ = 16.5, *df* = 1, *P* < 0.001). Mean bug abundance also declined significantly (Mann–Whitney test, *z* = 4.1, *P* < 0.001), and all infested houses were colonized (Fig. 3).

In rural areas, 11.1% of 45 previously infested houses that were re-sprayed at 1 YPS remained infested as determined by timed-manual searches 5 months later (May 2017). The prevalence of house infestation at 2 YPS reached 26.9%, with no significant reduction in mean bug abundance (Mann–Whitney test, *z* = −0.7, *P* = 0.5) relative to the preceding survey (Fig. 3, Table 2). Despite selective re-treatments with pyrethroid insecticide, 19.5% of the inspected dwellings were infested by timed-manual searches over 3–4 YPS, with 60.4% of them having been infested at least once during the follow-up. Mean bug abundance at 4 YPS was not significantly different from that recorded at 1 YPS (Mann–Whitney test, *z* = 0.6, *P* = 0.5).

**Fig. 4:**
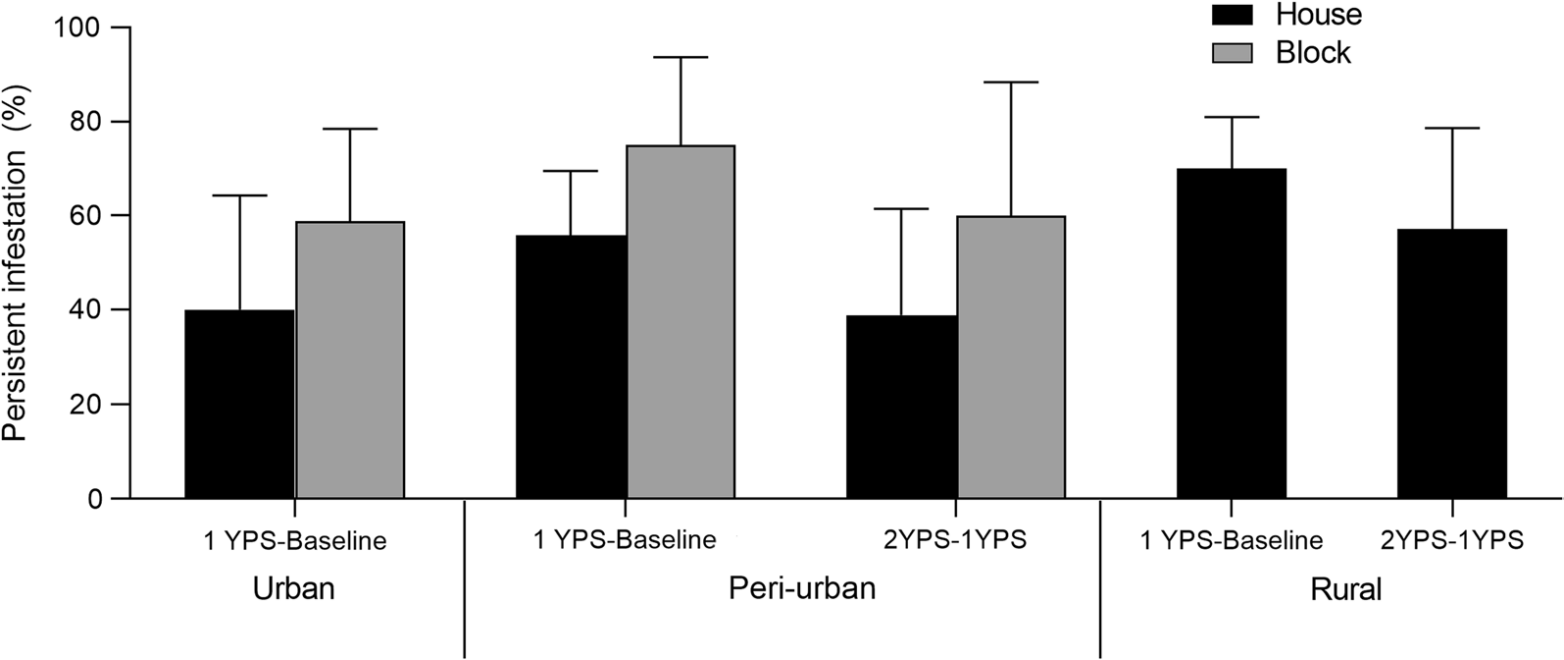
Persistence of house- and block-level infestation with *T. infestans*, as determined by timed-manual searches, according to intervention period and type of environment, Avia Terai, Chaco, 2015–2018

The frequency of infested houses that persisted between 0 and 1 YPS increased from 30.0 to 59.3% across the urban-to-rural gradient (Fig. 4). After selective re-treatment, similar levels of persistent infestation were registered in peri-urban (38.9%) and rural houses (57.1%). Block-level infestation persisted at greater levels in urban and peri-urban environments (Additional file 1: Text S1).

**Fig. 5:**
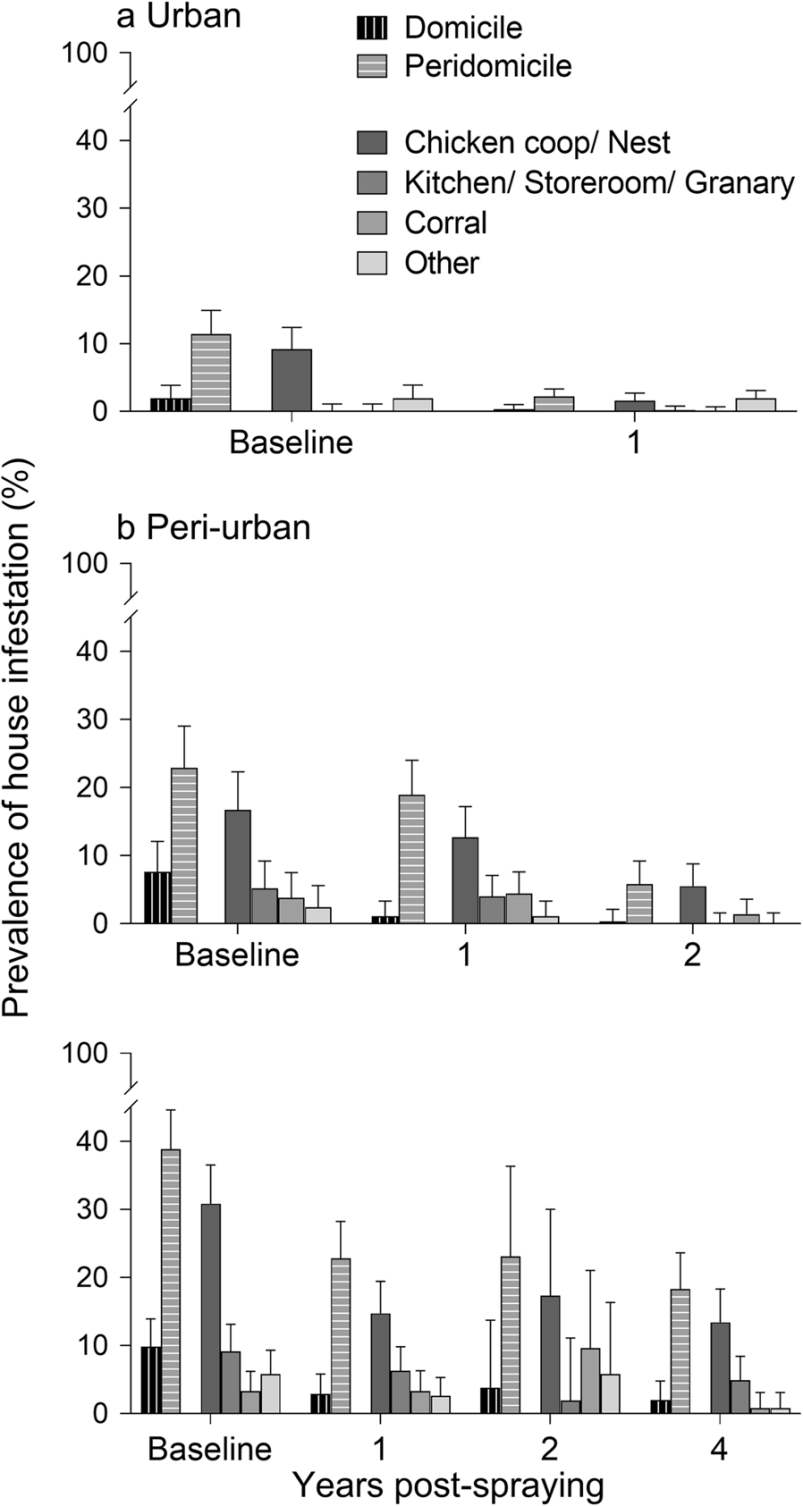
Ecotope-specific prevalence of house infestation with *T. infestans* according to environment (**a** urban; **b** peri-urban; **c** rural) during the follow-up, Avia Terai 2015–2019. ‘Other’ includes dog resting places, mud ovens, piled materials and collection sites with no data

Post-spraying house infestation was concentrated in peridomestic ecotopes with chickens associated across the urban-to-rural gradient, duplicating the pre-intervention pattern recorded in 2015–2016 (Fig. 5). Chicken-associated ecotopes accounted for 41.7, 62.5 and 61.5% of all infested houses at 1 YPS (as determined by any bug collection method) across the gradient, respectively. Infestation in other key peridomestic structures (such as kitchens, storerooms or corrals) retained a similar relative importance across environments over time. Domiciliary infestation at 1 YPS reached 8.3% and 12.3% of infested houses by any bug collection method in urban and rural areas, and 5.4% in peri-urban environments.

### Determinants of house infestation and bug abundance

Multiple logistic and negative binomial regressions showed that house infestation with *T. infestans* (by any collection method) and bug abundance (per one person-hour) at 1 YPS were 2.95–3.80 times higher if the house had been infested at baseline or was a high-risk house than in previously uninfested or low-risk houses, respectively (Table 4). Non-participating households at baseline had a 2.7 times higher relative odds of being infested than low-risk houses. House infestation and bug abundance in rural areas were significantly greater than in urban areas, but similar to those found in peri-urban houses. The only significant (negative) interaction involved non-inspected houses and the peri-urban environment. The Wald test was highly significant (*P* < 0.001). The Hosmer–Lemeshow test (*χ*^2^ = 7.37, *df* = 7, *P* = 0.36) and the area under the ROC curve (0.81) indicated a good fit of the logistic model to the data.

**Table 4:** Odds ratio (OR) and relative bug abundance (RA) for house infestation and bug abundance of *T. infestans* at 1 year post-spraying according to several determinants in Avia Terai, 2016–2017

Variable	Infestation		Bug abundance	
	OR	95% CI	RA	95% CI
Baseline infestation				
Not infested	1.00	–	1.00	–
Infested	3.31	1.99–5.53*	3.80	1.16–12.46*
Not inspected^a^	0.61	0.27–1.40	4.00	0.95–16.84
Initial house spraying				
Not sprayed	1.00	–	1.00	–
Sprayed	0.99	0.53–1.86	0.92	0.95–16.84
Baseline risk index				
Low-risk house	1.00	–	1.00	–
High-risk house	2.95	1.42–6.11*	23.81	5.27–107.59*
Non-participating^a^	2.73	1.16–6.39*	2.16	0.79–5.96
Environment				
Rural	1.00	–	1.00	–
Peri-urban	1.50	0.92–2.45	1.23	0.45–3.37
Urban	0.40	0.23–0.70*	0.31	0.12–0.79*

Number of houses, 1471

^a^Includes houses that were closed, refused access to premises, vacant or built after the baseline survey, in which the index could not be determined

*Statistically significant, *P* < 0.05

Peri-urban house infestation (by any collection method) at 2 YPS was only significantly and positively associated with baseline house infestation (Additional file 1: Text S1). Rural house infestation at 4 YPS was significantly higher in houses infested at 1 YPS or had high bug abundance at baseline (Additional file 1: Text S1).

Householder interviews revealed a few putative cases of passive transport of *T. infestans* from other infested houses during the follow-up. In one special case, pyrethroid-resistant *T. infestans* were collected at 1 YPS in a peri-urban house that had been non-infested at baseline. When asked on the putative origin of these triatomines, householders reported the recent introduction of roosters in cages from a house located in the same neighbourhood, where we subsequently collected pyrethroid-resistant triatomines.

### Pyrethroid resistance

**Fig. 6:**
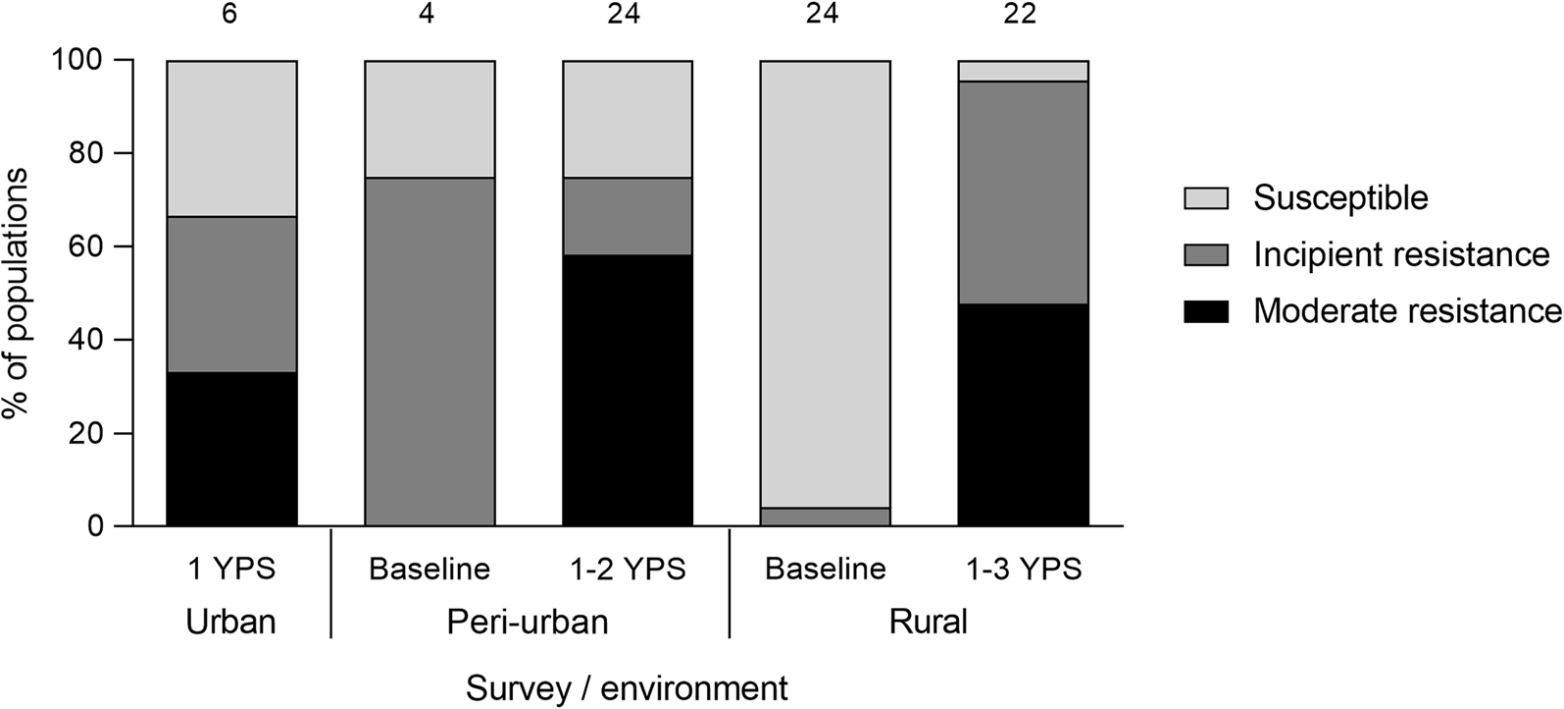
Distribution of deltamethrin resistance levels in *T. infestans* populations across environments, Avia Terai, 2015–2018. Susceptible, > 90% bug mortality; incipient resistance, 76–90% mortality; moderate resistance, 45–75% mortality. Numbers above bars indicate the number of individual triatomine populations (houses) tested

**Fig. 7:**
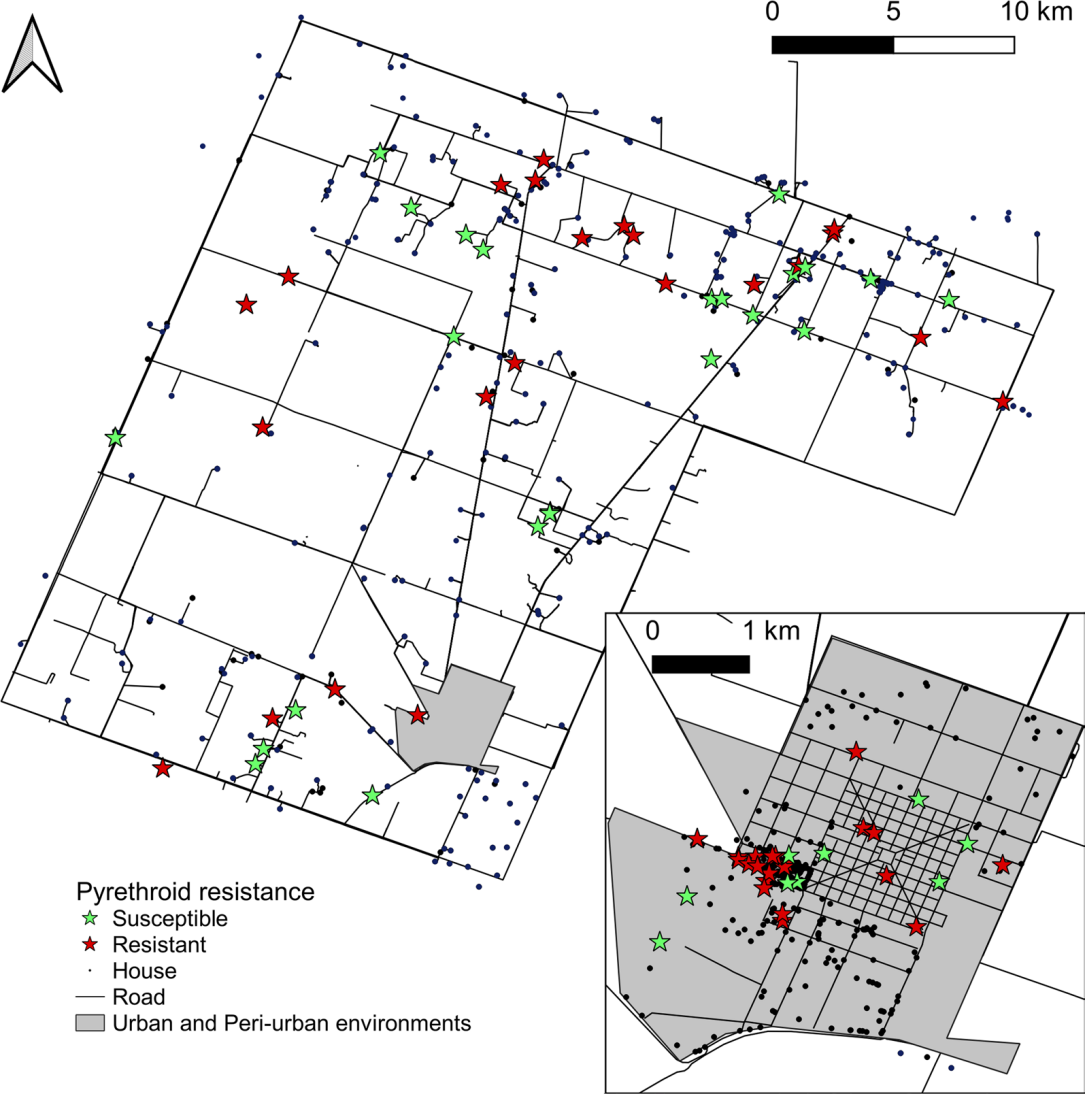
House-level distribution of pyrethroid-resistance status of *T. infestans* populations in **a** rural and **b** peri-urban or urban environments, Avia Terai, 2015–2018. Susceptible populations (green stars) had bug mortality > 90%; resistant populations (orange stars) showed mortality between 45 and 90% (incipient or moderate)

Two of the eight pools collected over 2015–2016 were resistant to deltamethrin. For triatomine populations screened at house level, only 7% (2 of 28) were deltamethrin-resistant before interventions conducted over 2015–2016, whereas 83% (43 of 52) of populations collected after insecticide spraying (2016–2018) were resistant; this difference was highly significant (Fisher’s exact test, *df* = 1, *P* < 0.001). Moderate pyrethroid resistance (i.e., bug mortality between 45 and 75%) occurred across the gradient over 2016–2018 (Fig. 6). Bug mortality never fell below 45%. Reduced bug mortality was detected in every infested rural or peri-urban community and throughout the urban blocks (Fig. 7). Five of six peri-urban triatomine populations with reduced bug mortality in response to the discriminating dose continued to show reduced mortality levels (57–75%) when treated topically with a five times greater concentration of deltamethrin.

Almost 60% of pre- and post-intervention samples screened for resistance had been collected in peridomestic sites with chickens associated. Resistant populations were detected in every ecotope over the follow-up, including those with chickens associated (58%, *n* = 48), kitchens/storerooms/granaries (60%, *n* = 15), domiciles (33%, *n* = 3), corrals (30%, *n* = 10) and other structures (piled materials, mud ovens, dog resting places) (60%, *n* = 5).

## Discussion

Our longitudinal study documents a complex pattern of persistence, fast recovery and conditional reduction of *T. infestans* populations over two periods following large-scale spraying with pyrethroid insecticide conducted by vector control personnel at a municipality-wide scale in the Argentine Chaco. Comparison of house infestation rates in 2011–2013 (first period) showed no significant reduction in house infestation across strata by 2015–2016. But when house infestation was compared at habitat level, domestic infestation decreased significantly while peridomestic infestation increased significantly across strata. Thus, the combined effects of partial spray coverage and non-systematic vector surveillance and response over the first period up to 2015 explain the limited impact of the first wave of interventions and determined that the prevalence of house infestation returned to pre-intervention levels within 3–4 years. A third factor, pyrethroid resistance, most likely played a relevant role as revealed during the second period.

One of our main goals was to assess whether the improved coverage of interventions combined with community mobilization (implying greater access to premises and spray coverage) exerted larger impacts on house infestation than preceding efforts during the first period. House infestation between 0 and 1 YPS relatively declined by 64%, with heterogeneous effects across strata. Unlike in the first intervention period, the decline in infestation occurred both at domestic and peridomestic habitat level. While overall insecticide spray coverage increased by 48% (from 722 to 1067 houses) from the first to the second period, spray coverage during the surveillance phase relatively increased by 154% (from 231 to 587 houses). The suite of interventions deployed over the second period exerted substantially larger effects on house infestation than the routine insecticide spraying campaign conducted earlier. The main obstacle to further progress was the occurrence of pyrethroid resistance and lack of alternative appropriate interventions rather than gaps in spray coverage and vector surveillance.

High-coverage house spraying with pyrethroids (reinforced with a double dose in peridomiciles) over the second period followed by selective treatments reduced both house infestation (though less than expected, i.e., < 5% within 1 YPS) and mean bug abundance across the urban-to-rural gradient over 0–1 YPS. The negative trend in mean bug abundance post-spraying is in line with detailed analyses of selected insecticide trials and triatomine control programs across the Argentine Chaco [44, Figs. 5 and 8]. Unlike in the latter, post-intervention infestation rates over 2–4 YPS remained near 20% in rural settings despite intervening insecticide applications. These patterns contrast with the either excellent or rather discrete outcomes of large-scale intervention trials of pyrethroids on the main domestic triatomine species (reviewed in [13]). Discriminating-dose bioassays (blind to field outcomes) showed the widespread occurrence of *T. infestans* foci with incipient or moderate resistance to pyrethroids. These findings most likely explain a large fraction of the post-intervention foci recorded, but does not disprove the occurrence of other sources of control failures, such as complex peridomestic structures (see below).

The emergence of non-trivial levels of pyrethroid resistance in Avia Terai was unexpected based on the sparse history of local insecticide applications and lack of proximity to high-resistance foci (the closest in Chaco were at approximately 150 km), including the occurrence of multiple susceptible triatomine populations in between [17]. Most triatomine populations tested at baseline (2015–2016) were susceptible to deltamethrin and other pyrethroids, as all deltamethrin-resistant populations of *T. infestans* were cross-resistant to other members in this insecticide class [16]. Of note, the frequency of tested triatomine populations that displayed incipient or moderate pyrethroid resistance substantially increased after the new insecticide spraying campaign conducted in 2015–2016, and the trend was verified across the gradient. These interventions selected for residual foci with reduced susceptibility to pyrethroids, and thus granted enhanced visibility to the underlying process. The larger impacts on house infestation in Avia Terai than in areas with high levels of pyrethroid resistance in Salta [41, 45] suggest that resistance levels were much lower in Avia Terai. First-instar nymphs of five triatomine populations that were treated topically with a five times greater discriminating dose of deltamethrin still displayed reduced mortality. Because pyrethroid-resistant *T. infestans* had much lower fertility and fifth-instar developmental rates than susceptible individuals [46], the former may propagate at lower relative rates when susceptible triatomines prevail.

Persistent infestations at house and community levels over both study periods (e.g., Figs. 2 and 4) imply residual foci (i.e., colonies in which a fraction of triatomines survived exposure to pyrethroids) rather than newly established infestations. Persistent foci usually display multiple late-stage nymphs [15]. Community-level house infestation rates over 2011–2013 and 2015–2016 were similar (Fig. 2). Additional evidence in support of the occurrence of residual foci is the positive relationship between post- and pre-intervention house infestation (or bug abundance) in the current study (Table 4, Additional file 1: Text S1) and elsewhere (i.e., [9, 47, 48]): the greater the number of bugs before insecticide spraying, the higher the infestation rates recorded soon after spraying. Moreover, no spatial aggregation of post-intervention infestation was detected (i.e., foci were randomly disseminated), unlike the epicentres recorded elsewhere [25, 49], and many triatomine populations were detected in rural houses 5 months post-spraying at 0 and 1 YPS. This set of convergent outcomes taken together with incipient or moderate pyrethroid resistance support the occurrence of control failures across the municipality and that the main driver of triatomine persistence was pyrethroid resistance. It is also consistent with the patterns recorded in Pampa del Indio (Chaco), where pyrethroid resistance unexpectedly emerged and was associated with control failures despite the lack of history of insecticide applications for triatomine control [15, 32]. A microsatellite-based study determined that a large fraction of post-spraying triatomines were genetically related to their pre-spraying counterparts collected at site level [50]. Even if passive transport and active dispersal of sylvatic *T. infestans* occurs marginally*,* they are unlikely to explain the large number of persistent foci detected after insecticide spraying.

The post-intervention follow-up confirmed the validity of the house risk index for rapid stratification purposes [34]. This index is based on the large positive predictive value of householder notifications of house infestation and the widespread occurrence of peridomestic infestations in rural areas (e.g., [11, 51]). Low-risk houses were not infested with *T. infestans* both before and after insecticide applications across the gradient. Non-participating households (mainly including closed, not accessible urban houses) also had an increased relative odds of being infested at 1 YPS, most likely because they had not been inspected and sprayed with insecticide (Table 4). This situation is partially analogous to that in Arequipa, Peru [28, 29]. Unlike in the latter, the fraction of Avia Terai households that refused to participate was minimal (1.3–4.0%), and the main reason for non-participation was absenteeism (closed house). Non-participating households and those with persistent infestations may serve as sources of triatomines for further propagation to other houses, more so in peri-urban or urban settings where houses are closer and lights attract flight-dispersing triatomines (e.g., [28, 52]). The risk index is especially helpful for prioritizing control actions in large-scale surveys and populated urban settings with low infestation rates, where the cost imposed by the large number of houses to inspect or spray is both demanding and difficult to justify.

House infestation increased across the urban-to-rural gradient over both periods, consistent with the pattern recorded before the second wave of interventions [34]. Peri-urban and rural settings had large infestation rates with *T. infestans* (20–40%) in the first period, when treatment coverage was well below target levels. Householders reported in 2015–2016 that 51–82% of houses had been sprayed with insecticides over 2011–2013 [34], thus providing additional evidence of partial spray coverage. The apparent high levels of urban house infestation over both periods (11–12%) may result from a sampling bias toward households or blocks that notified infestations (2011–2013) or targeted high-risk houses (2015–2016).

Triatomines were concentrated in peridomestic structures over time and space. The lower effectiveness of pyrethroids outdoors and in more exposed, frequently infested peridomestic structures such as chicken coops and corrals is a recurrent finding in the Argentine Chaco [9, 31, 32, 53, 54]. It is in part associated with type of construction materials, complex physical structure and insolation, which determine the fast decay of insecticide molecules and loss of residual lethality in peridomestic structures. This is a background cause of triatomine control failures regardless of pyrethroid resistance levels. Peridomestic house infestation recovered fast over the first period and even surpassed pre-intervention levels; declined between 0 and 1 YPS, and thereafter remained stable in rural areas. In contrast, domestic infestations (which were fewer and low-density) were substantially reduced after insecticide spraying, especially in urban and peri-urban houses, over both periods. This is relevant for parasite transmission since virtually all vector-mediated human infections with *T. cruzi* are acquired in domestic areas (human habitations). In Avia Terai, triatomine infection rates were notably low (< 2%) at baseline, and were virtually restricted to domestic areas and nearby structures used by dogs and cats [35]. With declining domestic infestations and triatomine abundance combined with already low bug infection rates (expected to further decline after insecticide spraying, [30]), the risk of transmission over the second period is minimal.

Some aspects of our longitudinal study limit the interpretation of results. The current assessment of intervention effectiveness comes from a pragmatic trial that relies on a before/after, stratified comparison, and therefore provides weaker evidence (including potentially biased effect measures) than a randomized controlled trial. The fact that program outcomes reflect the challenges of implementing district-wide interventions across an environmental gradient (affected by real-life constraints such as acceptability of interventions, weather, and availability of insecticide and field teams) is one of the strengths of this longitudinal study. Unfortunately, the combination of a strong El Niño event with other operational constraints did not allow us to assess the effects of block-level interventions on urban or peri-urban house infestations.

One major limitation of the first period was the absence of detailed census information to compute effective coverage rates; this was resolved in the second period and allowed us to gauge the fraction of house units not covered before. Lack of screening for pyrethroid resistance and estimates of bug abundance during the first period hindered a more detailed analysis of prior intervention effects on *T. infestans* populations. Screening of a substantial number of (pooled or individual) triatomine populations across the municipality before and after the second wave of interventions provided robust evidence of pyrethroid resistance regardless of the exact number of houses contained in resistant pools. (If all houses in a resistant pool had been considered resistant, then only 2–4 houses per pool would have shifted its status.) Further efforts to determine the intensity of pyrethroid resistance and the underlying mechanisms and to map its occurrence are needed.

Any assessment of the outcome of triatomine control interventions is affected by the limited sensitivity of available sampling methods. Although timed-manual searches (with or without a dislodging aerosol) have historically been the reference method, their sensitivity is influenced by the ecotopes’ physical structure and triatomine density, among other factors [55, 56]. Consequently, the observed infestation levels are expected to underestimate the true levels by an undefined factor. Repeated searches over time would mitigate this limitation at the expense of additional labour costs; this may not be feasible in large-scale studies such as the current one. Community-based triatomine surveillance was meant to provide additional evidence, and has worked effectively in Avia Terai and elsewhere [11, 34, 51, 57]. Timed-manual searches and householder notifications revealed 70% and 54% of actual house-level infestations detected by a range of methods, respectively [58]. Their combined outcome would roughly reveal 80% of all house infestations detected, more so in domestic habitats (87%); thus, the observed prevalence of infestation adjusted for imperfect detection would increase relatively by 15–25% and would hardly affect the qualitative conclusions in this paper.

Future research efforts should assess the cost-effectiveness of both sets of field interventions. More specifically, what are the costs of failing to provide a full-coverage insecticide spraying of high quality during the attack phase in terms of residual (re)infestation, parasite transmission and future costs of launching renewed interventions. The combined cost of transportation, vehicle maintenance and per diems of spray teams almost tripled the total cost in insecticide when two rounds of residual spraying with pyrethroids were applied by a vertical control program of *T. infestans* in the dry Argentine Chaco [31]. Failure to treat and suppress most triatomine foci during the attack phase imply greater reinfestation rates and chances of reaching the 5% house-infestation threshold that in theory should lead to launching a new attack phase with its attached costs.

## Conclusions

Residual house spraying with pyrethroid insecticide has successfully suppressed or reduced house infestations with the major triatomine species over the last 40 years, and continues to be effective under most circumstances if applied properly in the framework of a consistent disease control program [13]. A single insecticide application is rarely able to entirely suppress house infestation with triatomines at a community-wide level; vector surveillance and response is key to the success of control operations. This is especially important in the frame of intensified efforts to diagnose and treat *T. cruzi*-infected patients living in traditionally endemic areas, where lack of effective vector surveillance increases the risk of re-infection [59].

Vector control failures are increasingly reported in the Gran Chaco, and may be attributed to two main processes: poor implementation of vector control operations (e.g., substandard coverage and faulty technical procedures, inconsistent vector surveillance and response) and pyrethroid resistance [13]. Distinguishing between both mechanisms is essential for an appropriate prescription of control actions. Standard good practices include monitoring of the quality of interventions, their effects, and the eventual occurrence of pyrethroid resistance. For example, the pyrethroid resistance status of *T. infestans* populations collected in Avia Terai over 2011–2013 had not been determined. Quality assurance procedures need to be applied systematically and more widely. Suspect vector control failures should be investigated rigorously, and remedial measures effected. Evidence-based vector control and surveillance interventions adapted to local settings is key to improving and/or sustaining the achievements [60]. Alternative insecticides to suppress pyrethroid-resistant triatomine populations are greatly needed, as are evidence-based intervention protocols that can deal with urban infestations in a cost-effective way (e.g., [61]).

In addition to *T. infestans*, other triatomine species have displayed reduced susceptibility to pyrethroids elsewhere, including *Rhodnius prolixus* from Venezuela [62] and Colombia [63], *T. sordida* from Minas Gerais [64], and *Triatoma mazzotti* and *Triatoma longipennis* from Mexico [65]. Whether such reduced susceptibility levels actually cause control failures is a key question across triatomine species and settings, and entail linking the outcomes of discriminating dose bioassays to village-level house infestation patterns. Whether the steady expansion of the agricultural frontier, as occurred in Avia Terai, and the associated intensified use of agrochemicals might be related to the emergence of pyrethroid resistance in *T. infestans* remains to be determined.

One of the immediate goals of Chagas disease control programs is the interruption of vector-borne transmission via suppression or strong reduction of domestic triatomine populations. Although the elimination of *T. infestans* was not achieved in Avia Terai, the set of interventions substantially reduced domestic bug abundance and virtually restricted infestation to peridomestic structures with chickens associated (where bug infection with *T. cruzi* is typically nil). Consequently, the risk of human infection with *T. cruzi* most likely plummeted and may remain so unless domestic recolonization proceeds. The sustainable elimination of *T. infestans* from highly endemic scenarios such as those in the Gran Chaco requires consistent efforts ideally framed in integrated vector management principles with strong engagement of local communities, diagnosis and etiological treatment. Housing improvement and environmental management of peridomestic structures have been notoriously neglected so far, and may play a crucial role for sustainable vector elimination.

## Supplementary Information

**Additional file 1: Text S1.** Potential urban sampling bias, urban and peri-urban block-level infestation and determinants house infestation from 2 YPS onward, Avia Terai, 2016–2019.

**Additional file 2: Figure S1.** Spatial distribution of house infestation with *Triatoma infestans* in rural houses, peri-urban houses and blocks, and urban blocks at baseline (**a**), 1YPS (**b**), 2 YPS (**c**), 4 YPS (**d**), Avia Terai, 2016–2017.

**Additional file 3: Table S1.** Individual house data including entomological variables and pyrethroid resistance during the follow-up, Avia Terai, 2015–2019.

**Additional file 4: Table S2.** Block-level data including entomological variables and pyrethroid spraying during the follow-up, Avia Terai, 2015–2018.

**Additional file 5: Table S3.** Individual house data including entomological variables and pyrethroid spraying during the first period, Avia Terai, 2011–2013.

## Data Availability

The datasets generated during and/or analysed during the current study are available as additional files (Additional file 3: Table S1, Additional file 4: Table S2, Additional file 5: Table S3).

## Abbreviations

ROC: Receiver operating curve
CMH: Cochran–Mantel–Haenszel
YPS: Years post-spraying
