# Supplementary material for: Improved vector control of Triatoma infestans limited by emerging pyrethroid resistance across an urban-to-rural gradient in the Argentine Chaco
Source: Parasit Vectors. 2021 Aug 28;14:437. doi: 10.1186/s13071-021-04942-9 (PMC8401064; doi:10.1186/s13071-021-04942-9)
Supplement: Supplementary file 1 — Additional file 1: Text S1. Potential urban sampling bias, urban and peri-urban block-level infestation and determinants house infestation from 2 YPS onward, Avia Terai, 2016–2019. [file 13071_2021_4942_MOESM1_ESM.docx]

**Additional File 1: Text S1**

Potential sampling bias in urban areas

Comparison of house infestation prevalence between 2011-2013 and 2015-2016 significantly declined from 11.8% to 4.5% (χ^2^ = 17.52, *df* = 1, *P* < 0.001) when the projected prevalence obtained from the latter survey was considered. The projected prevalence of infestation was assessed considering the number of infested houses, the risk status of each not-inspected house, and the total number of inhabited houses in the environment (Gaspe et al. 2020). If triatomine search efforts in urban settings during the first period had been biased to households that notified infestations or which had a higher probability of infestation, a more valid comparison of house infestation prevalence between both surveys would be with the observed prevalence of infestation during the second period as shown in the main text.

Block-level infestation in urban and peri-urban areas

The impact of insecticide spraying on urban block infestation was lower than expected: it declined from 27.8% at baseline to 16.8% at 1 YPS, with 55.6% of infested blocks showing a persistent infestation. Similarly, in established peri-urban neighborhoods, block infestation slightly dropped from 64.3% to 57.1 %, and 75% of infested blocks as of 1 YPS had been infested at baseline. High persistence at block level prompted the application of insecticide to all houses in an urban or peri-urban block that had at least an infested house at 1 YPS. Peri-urban block infestation displayed no significant change one year later (2 YPS) (from 57.1 to 35.7%) (Fishers’ exact test, *df* = 1, *P* = 0.4). As urban houses were not re-surveyed at 2 YPS, we could not assess the impacts of changing the spray coverage protocol at block level.

Urban block infestation and the number of infested houses in a block at 1 YPS (response variables) were associated positively and significantly with the number of high-risk houses registered in the block at baseline using multiple logistic and negative binomial regressions (OR: 1.46, 95% CI: 1.15-1.85, n = 92; OR: 1.28, 95% CI: 1.11-1.47, n = 107, respectively). No significant effects of block infestation at baseline or the number of houses sprayed in a block were detected. The logistic model showed a good fit according to the Hosmer-Lemeshow test (*χ*^2^ = 6.26, *df* = 6, *P* = 0.39) and the area under de ROC curve was 0.79.

Determinants of house infestation from 2 YPS onward

Using multiple logistic regression analysis, peri-urban infestation at 2 YPS was 6.6 times higher if the house had been infested at baseline rather than not (OR: 6.60, 95% CI: 1.94 - 22.51, n = 240), but no significant effect of prior infestation at 1 YPS was detected (OR: 1.97, 95% CI: 0.65–5.96). Both the Hosmer-Lemeshow test (*χ*^2^ = 0.55, *df* = 3, *P* = 0.91) and the area under de ROC curve (0.77) indicated a good fit to the data.

Rural infestation at 4 YPS was 3.66 times higher if the house had been infested at 1 YPS survey (OR: 3.66, 95% CI: 1.23–10.91), but no association was found with house infestation at baseline or at 2YPS (model 1). In a second model, house infestation at 4 YPS was almost three-fold higher in houses with abundant triatomines at baseline (OR: 2.47, 95% CI: 1.01–6.01), and no significant effects were detected at lower levels of bug abundance at baseline, or with bug abundance at 1 YPS or house infestation at 2 YPS (model 2). Both models for house infestation at 4 YPS showed good fit to the data as measured by Hosmer-Lemeshow tests (model 1: *χ*^2^ = 1.18, *df* = 4, *P* = 0.88; model 2: *χ*^2^ = 3.76, *df* = 5, *P* = 0.58) and the area under the ROC curve (model 1: 0.66; model 2: 0.70).
