# Supplementary figures and images for: Improved vector control of Triatoma infestans limited by emerging pyrethroid resistance across an urban-to-rural gradient in the Argentine Chaco
Source: Parasit Vectors. 2021 Aug 28;14:437. doi: 10.1186/s13071-021-04942-9 (PMC8401064; doi:10.1186/s13071-021-04942-9)

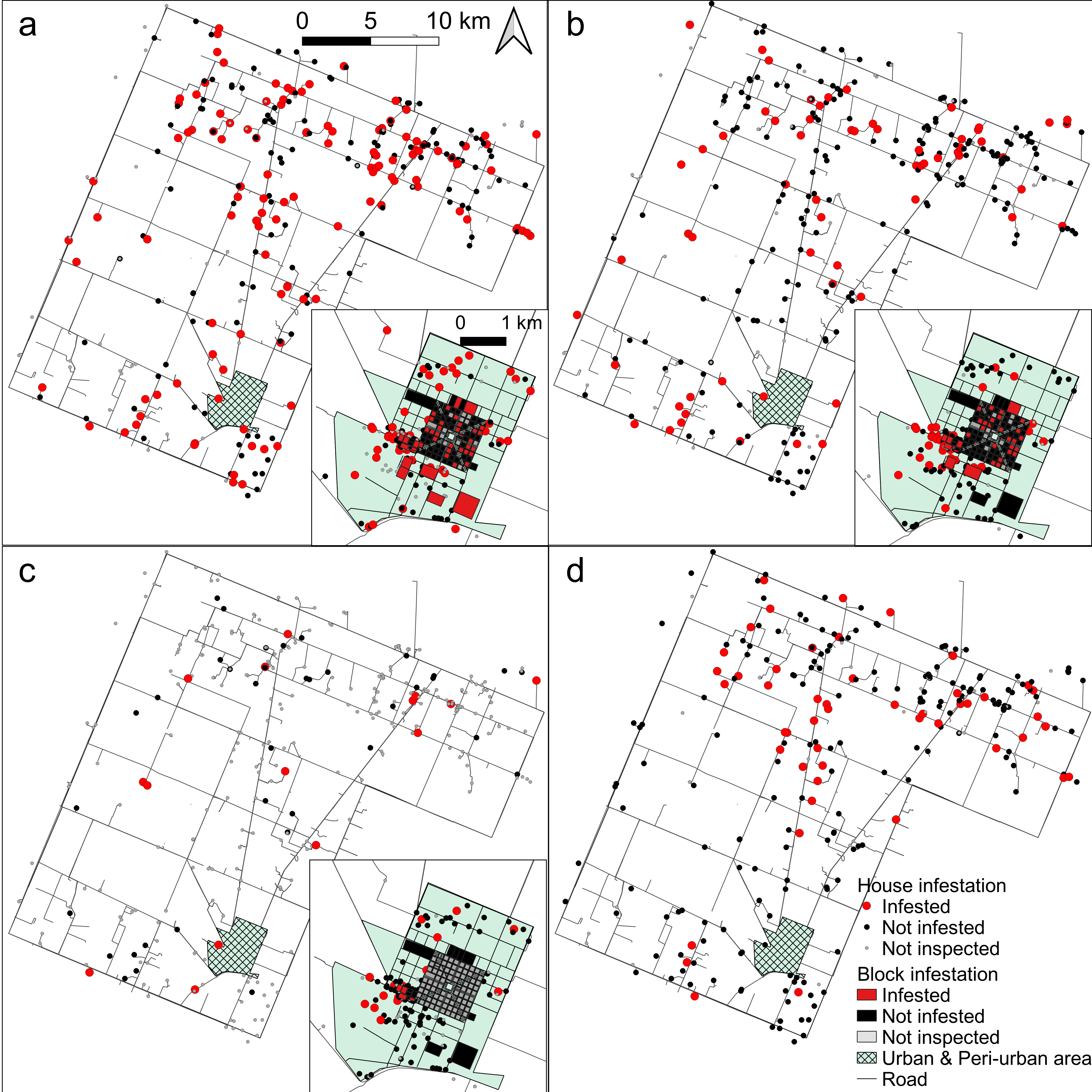

Supplement: Supplementary file 2 — Additional file 2: Figure S1. Spatial distribution of house infestation with Triatoma infestans in rural houses, peri-urban houses and blocks, and urban blocks at baseline (a), 1YPS (b), 2 YPS (c), 4 YPS (d), Avia Terai, 2016–2017. [file 13071_2021_4942_MOESM2_ESM.tif]
